# PP34 Impact Of The COVID-19 Pandemic On Depression Incidence And Healthcare Service Use Among Patients With Depression

**DOI:** 10.1017/S026646232400206X

**Published:** 2025-01-07

**Authors:** Vivien Kin Yi Chan, Yi Chai, Sandra Sau Man Chan, Hao Luo, Mark Jit, Martin Knapp, David Makram Bishai, Michael Ni, Ian Chi Kei Wong, Shirley Xue Li

## Abstract

**Introduction:**

Most studies on the impact of the COVID-19 pandemic on depression burden focused on the earlier pandemic phase specific to lockdowns, but the longer-term impact of the pandemic is less well studied. In this population-based cohort study with quasi-experimental design, we examined both the short-term and long-term impacts of COVID-19 on depression incidence and healthcare service use among patients with depression.

**Methods:**

Using the territory-wide electronic medical records in Hong Kong, we identified patients with new diagnoses of depression from 2014 to 2022. An interrupted time-series (ITS) analysis examined changes in incidence of depression before and during the pandemic. We then divided patients into nine cohorts based on year of incidence and studied their initial and ongoing service use until December 2022. Generalized linear modeling compared the rates of healthcare service use in the year of diagnosis between patients newly diagnosed before and during the pandemic. A separate ITS analysis explored the pandemic impact on the ongoing service use among preexisting patients.

**Results:**

There was an immediate increase in depression incidence (RR=1.21; 95% CI: 1.10, 1.33; p<0.001) in the population since the pandemic with a nonsignificant slope change, suggesting a sustained effect until the end of 2022. Subgroup analysis showed that increases in incidence were significant among adults and the older population, but not adolescents. Depression patients newly diagnosed during the pandemic used 11 percent fewer resources than the prepandemic patients in the first diagnosis year. Preexisting depression patients also had an immediate decrease of 16 percent in overall all-cause service use since the pandemic, with a positive slope change indicating a gradual rebound.

**Conclusions:**

During the COVID-19 pandemic, service provision for depression was suboptimal in the face of greater demand generated by the increasing depression incidence. Our findings indicate the need to improve mental health resource planning preparedness for future public health crises.

